# Consumer responses to rebranding to address racism

**DOI:** 10.1371/journal.pone.0280873

**Published:** 2023-02-08

**Authors:** Maria Kalaitzandonakes, Brenna Ellison, Tiffany White

**Affiliations:** 1 Department of Agricultural & Consumer Economics, University of Illinois Urbana-Champaign, Champaign, Illinois, United States of America; 2 Department of Agricultural Economics, Purdue University, West Lafayette, Indiana, United States of America; 3 Gies College of Business, University of Illinois Urbana-Champaign, Champaign, Illinois, United States of America; Universidad de Los Andes, COLOMBIA

## Abstract

In 2020, following the death of George Floyd and the renewed national focus on racism, many food brands with racist names and packages announced they would rebrand. Brands differed in their extent of rebranding (some only removed an image, whereas others also changed a brand name) and differed in the reasons they gave for the rebranding in PR statements and news interviews. At this point, little is known about how consumers responded to these branding changes. To address this, we conducted an online experiment using the case of Aunt Jemima pancake mix to evaluate how changes in the extent of rebranding and the reason for rebranding impact consumers’ likelihood of purchase, expected taste, brand liking, and brand trust. We find that removing the image of Aunt Jemima brought moderate reductions to likelihood of purchase and expected taste and no changes to brand liking or brand trust. When the brand name was also changed to Pearl Milling Company we find larger reductions to likelihood of purchase and expected taste and reductions to brand liking and brand trust. Additionally, we find that informing consumers the change was done to address racism partially mitigated losses in likelihood of purchase following renaming the brand but provided no protection when only the image was removed. The information also had no impact on expected taste, brand liking, or brand trust following either image removal or brand name change. Last, we find evidence of heterogeneity in consumer responses across political ideologies, with liberals reacting more positively to the rebranding and conservatives reacting more negatively.

## Introduction

Following the death of George Floyd in 2020 and subsequent Black Lives Matter protests, food companies began announcing plans to remove racist images on packaging and plans to change racist brand names, including Eskimo Pie (now Edy’s Pie) [1], Mrs. Butterworth [2], Uncle Ben’s (now Ben’s Original) [3], and Cream of Wheat [2]. Calls for these food companies to rebrand were not new, as consumers’ and advocacy groups’ demands date back decades (e.g., [4]), but companies had long avoided it. Companies spend large amounts of money to create and maintain brands, and marketing literature has shown that brands become valuable long-term assets. For consumers, brands can serve as indicators of quality, reduce risk in purchasing, and speed up frequent decision making–all of which can increase willingness to pay (e.g., [5–7]). Additionally, consumers can more easily process images they are more familiar with (e.g., a familiar brand), and this can cause consumers to regard the image as “correct” or “how it should be,” resulting in higher liking (e.g., [8]). Reduced brand recognition through rebranding, therefore, could be costly, and the process of redoing and rolling out new packaging is also expensive [9]. However, a brand’s ties to racism and the potential losses due to negative PR or boycotts could also be costly [10]. Rebranding in any form is therefore a major decision for a company. Reviews of the rebranding literature show that these food companies were rebranding under unique circumstances. Rebranding is most often slowly planned by the company and generated by internal plans, rather than motivated by external criticism and accompanied by a hurried timeline, as was the case here [11]. Additionally, renaming a brand is most often due to a structural change (e.g., merger) and is often accompanied by a large advertising push highlighting the continuity of the new name to the old brand [12], which could be more difficult in this case.

At this point, there is little research that evaluates consumer responses to brand racism and, to our knowledge, no research that evaluates how consumers react when companies rebrand to address racism. Most closely related is Miller, Stanko, and Diallo’s overview of potential actions companies can take when their brand is racist [10]. The authors highlight issues that companies may need to address during rebranding, including: a reduction in brand recognition, consumers believing the product itself has also changed, and announcements bringing additional attention to bad attributes. Other relevant papers have looked at how consumers respond via social media after brands are associated with racism. For example, Wei and Bunjun evaluate Twitter responses to New Balance shoes after the brand was associated with, and then publicly rejected, white supremacy [13]. Research on consumer responses to brands’ actions with other divisive topics is also relevant. For example, researchers have evaluated US consumers’ changes in purchasing of products with “French-sounding” names following French opposition to the Iraq War [14], changes in US consumers’ purchasing of Goya products during a politically charged boycott and buycott [15], and changes in Mexican consumers’ purchasing of American coffee brands following the proposal to build a wall on the countries’ border [16].

This paper utilizes the case of Aunt Jemima pancake mix to evaluate consumer responses to rebranding to address racism. The brand has used the Aunt Jemima name and image since the 1890s and hired Nancy Green, a former slave, to portray the Aunt Jemima character [4]. Research on the company’s advertising decisions notes, “it is widely acknowledged that the historical basis for the Aunt Jemima trademark is the plantation slave of the antebellum South known as ‘Mammy.’” [17]. The brand was purchased by Quaker Oats in 1925, which was acquired by PepsiCo in 2001 [18]. The image of Aunt Jemima has been through several updates over time, in an effort to distance the image from its original imagery (e.g., switching her bandana to a headband) [17]. Most recently, in 2020 and 2021, PepsiCo removed the image of Aunt Jemima and renamed the brand Pearl Milling Company. In the first stage of rebranding the company removed only the image of Aunt Jemima from its packaging and in the second stage of the rollout, the company also changed the brand name to Pearl Milling Company.

Food companies differed in the extent of rebranding. Some food brands, like Aunt Jemima, changed their name and removed imagery from their packages (e.g., Uncle Ben’s removed an image and changed their name to Ben’s Original [3]), however some food companies only adjusted imagery (e.g., Cream of Wheat [2]). How food companies spoke about their reasons for rebranding in public announcements and news articles also differed, with some noting directly that the change was to address racism and others not. Some also announced accompanying donations (e.g., [3]).

In this paper, we test the differential impact of the extent of rebranding and the reason for rebranding on likelihood of purchase, expected taste, brand liking, and brand trust using a 2x3 pre/post design. All participants rated the dependent measures for both the original (pre-rebranding) and rebranded product (post-rebranding). We varied the extent of rebranding (2: Image Removal Only or Image Removal & Name Change) and the reason for rebranding (3: Racism Information, Racism & Donation Information, or Alternative Information). To vary extent, participants were randomly assigned to see one of the two versions of the rebranded Aunt Jemima product, which allowed for a clean comparison as the product itself did not change. To vary the reason for rebranding, participants were randomly assigned to see text that indicated the reason for rebranding. We use Difference in Differences (DID) methodology to test whether responses to rebranding to address racism differed from rebranding for an alternative reason.

We find that removing the image was associated with an 8% decrease in likelihood of purchase and informing participants that the image was removed to address racism in the brand and packaging did not offer any protection over an alternative reason. When the brand name was also changed there was a larger drop in likelihood of purchase, a loss of about 32%, and informing participants that the renaming was done to address racism in the brand and packaging provided a partial buffer, increasing likelihood of purchase by about 10% over an alternative reason. Additionally, indicating that the brand had added a $5 million donation to support the Black community did not increase likelihood of purchase over the racism information alone. We find evidence that consumers believe the product itself has changed, as expected taste drops following both the removal of the image only and renaming the brand. Brand liking and brand trust were not affected by the image removal, however, both were reduced when the brand name changed. Informing consumers that the rebranding was done to address racism did not mitigate losses in expected taste, brand liking, or brand trust over an alternative reason.

Additionally, we investigate heterogeneity across political ideology, as previous literature indicates responses could be politically divisive. For example, research has found that consumers with different political ideologies feel differently about calling out potentially offensive content, where liberals think this holds people accountable and conservatives believe it punishes people [19]. These perceptions may extend to how consumers feel about brand actions. More broadly, beyond the more utilitarian value consumers receive from consuming or using a product, consumers can also receive benefits of “warm glow” associated with satisfaction from doing good [20] and benefits from engaging in acts as political consumers [21]. Previous research, for example, documented how consumers from different political parties engaged in boycotts/buycotts of Goya Foods as a short-term act of political consumerism [15]. Finally, there are large differences in support surrounding the protests which occurred during the time of the rebranding announcements. Pew Research Center found a very large gap between Republican and Democrat support for Black Lives Matter, 19% and 85% respectively [22]. We find that although the effect of information was often small or null on average, responses differed considerably across political ideology. In general, the racism information seemed to buffer some of the losses for both liberals and moderates (with larger increases for liberals) but work in reverse for conservatives–with larger losses under racism information treatments than under the alternative information treatment.

Although this paper focuses on the food industry, specifically Aunt Jemima pancake mix, this discussion goes well beyond it–from sports (e.g., Washington Redskins, now the Washington Commanders, Cleveland Indians, now the Cleveland Guardians, Kansas City Chiefs [23]) to bands (e.g., Lady Antebellum, now Lady A and The Dixie Chicks, now the Chicks [24]) to consumer products more broadly (e.g., Darlie toothpaste, now Haolai, and Fair & Lovely cream, now Glow & Lovely [25]). This issue is also not limited to large companies. Smaller brands across the country are also reevaluating their names and branding (e.g., Dixie Brewery, now Faubourg Brewing Co. [10]). Additionally, although we focus on racism here, this is only becoming more relevant broadly, as consumers become more vocal about the behavior of brands on a wide range of divisive issues.

## Survey & experimental design

We conducted an online experiment with 1,506 US consumers using CloudResearch panels that was designed to mirror the US population in terms of sex, age, and geographic region. The experiment was conducted in November 2021. Our sample characteristics reflect the US population across sex, region, age, and race quite well. However, our sample underrepresents the Hispanic/Latino population and those with annual household income above 100,000 and overrepresents those with income less than $50,000. Our sample also slightly overrepresents moderates and underrepresents conservatives compared to 2020 estimates [26]. Table 1 provides a summary of participant characteristics.

**Table 1:** Characteristics of sample.

Characteristic	Description	Percent of Sample
**Sex**	Male	48.80%
	Female	51.20%
**Age**	18–24 years	12.95%
	25–34 years	17.46%
	35–44 years	17.86%
	45–54 years	19.06%
	55–64 years	15.41%
	65+ years	17.26%
**Region**	Northwest	18.46%
	Midwest	21.78%
	South	37.05%
	West	22.71%
**Race**	White	76.69%
	Black or African American	12.95%
	Other	10.36%
**Ethnicity**	Hispanic/Latino	10.96%
	Not Hispanic/Latino	89.04%
**Education**	Less than high school	4.05%
	High school/GED	28.88%
	Some college	23.71%
	Associates or technical degree	12.62%
	Bachelor’s degree	19.39%
	Graduate or professional degree	11.35%
**Annual Household Income**	Less than $25,000	26.76%
	$25,000–$49,999	29.35%
	$50,000–$74,999	17.26%
	$75,000–$99,999	10.76%
	$100,000 +	15.87%
**Political Ideology**	Liberal	25.23%
	Moderate	46.88%
	Conservative	27.89%

This study was approved by the University of Illinois at Urbana-Champaign Institutional Review Board (IRB #22308). Respondents provided written consent by answering affirmatively that they would like to participate in the study in the first question of the online survey. After providing consent, all participants first answered questions on dependent measures (likelihood of purchase, expected taste, brand liking, and brand trust) for the original Aunt Jemima packaging (*t*_0_). Participants were then randomized into one of six treatment groups using a 2x3 design (see Table 2), where we varied the extent of rebranding (2: Image Removal Only or Image Removal and Name Change) and the reason for rebranding (3: Racism Information, Racism & Donation Information, or Alternative Information). Finally, participants answered questions on dependent measures again for one of the rebranded packages (*t*_1_).

**Table 2:** Experimental design.

Within Subject	Between-Subjects					
Original packaging (*t*_0_)	All participants					
Rebranded packaging (*t*_1_)	Image Removal Only			Image Removal & Name Change		
	Racism Info	Racism & Donation Info	Alternative Info	Racism Info	Racism & Donation Info	Alternative Info
	Group 1	Group 2	Group 3	Group 4	Group 5	Group 6

We utilized Aunt Jemima’s two-stage rollout to vary the extent of rebranding. Half of the participants were randomly assigned to evaluate the rebranded package where only the image of Aunt Jemima was removed (Image Removal Only) and half were randomly assigned to evaluate the rebranded package where the name of the product was also changed to Pearl Milling Company (Image Removal & Name Change). Each participant was also randomly assigned to read one of three information treatments that explained the reason for rebranding before viewing the updated product. The information treatments were short news excerpts we wrote to reflect the variety of responses by food companies following rebranding. The news excerpts included AdWeek as the source to avoid an association with a particular political ideology. One information treatment (Racism Information) indicated that the rebranding was done to address racism in the brand and packaging. A second information treatment (Racism & Donation Information) added an additional line to the first treatment that indicated the company had pledged $5 million to support the Black community, which reflects the donation amount pledged by PepsiCo. Finally, we included an alternative reason for the rebranding (Alternative Information) that indicated the change was “to increase interest in their brand and packaging.” We measure the impact of the Racism Information and Racism & Donation Information beyond the impact of the Alternative Information. This allows us to evaluate whether informing participants the rebranding was done to address racism was associated with changes beyond rebranding for a more typical reason to rebrand. Full information treatments are available in S1 Table. Knowledge checks following the information treatments verified that participants had read and understood the reason for and extent of rebranding.

Likelihood of purchase was measured on a scale from 1 (not very likely to buy) to 10 (very likely to buy). Expected taste was also measured on a scale from 1 (tastes bad) to 10 (tastes good). We assessed brand liking using a four-item scale [27] from 1 (unappealing, bad, unpleasant, unlikeable) to 7 (appealing, good, pleasant, likeable) and then averaged the scores for a single measure of brand liking. Similarly, we assessed brand trust using a four-item scale from 1 (not trustworthy, not reliable, dishonest, not credible) to 7 (trustworthy, reliable, honest, credible) and averaged each participants’ responses for a single brand trust score.

## Data analysis

We use Difference in Differences (DID) to evaluate the impact of rebranding and the reason for rebranding on our dependent variables, outlined in Eq 1.

$$y_{ist}=\alpha_{0}+\beta *Post_{t}+\gamma_{1}*R\;Info_{s}+\gamma_{2}*R\&D\;Info_{s}+\delta_{1}*{\left(Post\times R\;Info\right)}_{st}+\delta_{2}*{\left(Post\times R\&D\;Info\right)}_{st}+\varepsilon_{ist}$$ (1)

where *y*_*ist*_ is the dependent variable (likelihood of purchase, expected taste, brand liking, or brand trust) for individual *i* in treatment group *s* in time period *t*. *Post* takes the value of 0 when participants viewed the product prior to rebranding (*t*_0_) and 1 when the participant viewed the rebranded product (*t*_1_). *R Info* takes the value of 1 when participants were randomly assigned to view the Racism Information treatment and 0 otherwise and *R* & *D Info* takes the value of 1 when participants were randomly assigned to see the Racism & Donation Information treatment and 0 otherwise. Both are compared the Alternative Information treatment. *β* accounts for the first difference, the impact of rebranding; *γ* accounts for group level differences; and *δ* accounts for the second differences, the impact of the information treatments over the alternative reason. We chose to use an alternative reason, rather than no information, to ensure our results would not be driven by attention to the product or awareness of rebranding. We conduct the analysis for the rebranded product after only the image of Aunt Jemima was removed (Image Removal Only) and the rebranded product after both the image was removed and the brand name was changed to Pearl Milling Company (Image Removal & Name Change) separately for each dependent variable.

Additionally, to look at differences across political ideology more closely, we calculate the change in each dependent variable for each participant (*i*),

$$\Delta y_{ist}=y_{ist_{1}}-y_{ist_{0}}$$ (2)

and then compare the average change in likelihood of purchase, expected taste, brand liking, and brand trust across the six treatments and political ideology. We test for differences in political ideology for each treatment group using ANOVA tests. For treatment groups with significant differences across political ideologies, we use t-tests to compare differences between each pair of political ideologies.

## Results

### Main results

Table 3 compares means of likelihood of purchase, expected taste, brand liking, and brand trust across treatment groups prior to and following rebranding. We find that the established Aunt Jemima brand had high initial levels of likelihood of purchase (between 7.60 and 8.06 on a 10-point scale), indicating this is a product the average consumer is likely to buy. We also see that initial values of expected taste, brand liking, and brand trust were high. Average initial values of expected taste ranged between 8.77 and 8.94 (on a 10-point scale) across groups. Average initial values of brand liking and brand trust were 5.41 to 5.63 and 5.18 to 5.44 (on a 7-point scale) across groups, respectively.

**Table 3:** Mean measures across groups prior to (*t*_0_) and following rebranding (*t*_1_).

	Image Removal Only			Image Removal & Name Change		
	Racism Info	Racism & Donation Info	Alternative Info	Racism Info	Racism & Donation Info	Alternative Info
Likelihood of Purchase						
t_0_	8.06	7.60	7.77	7.92	7.80	7.91
t_1_	7.29	6.95	7.14	6.16	5.91	5.39
Expected Taste						
t_0_	8.84	8.77	8.91	8.94	8.85	8.80
t_1_	8.43	8.45	8.54	7.51	7.35	7.20
Brand Liking						
t_0_	5.51	5.42	5.54	5.57	5.41	5.63
t_1_	5.16	5.15	5.32	4.44	4.45	4.32
Brand Trust						
t_0_	5.25	5.18	5.44	5.36	5.26	5.38
t_1_	5.29	5.21	5.41	4.51	4.57	4.43

*Note*: Likelihood of purchase and expected taste were measured on a scale from 1 (low) to 10 (high). Brand liking and brand trust were measured on a scale from 1 (low) to 7 (high).

Table 4 presents the DID results for likelihood of purchase, expected taste, brand liking, and brand trust. We find high initial values for likelihood of purchase, expected taste, brand liking, and brand trust for the brand (*α*_0_) and no significant differences across treatment groups (*γ*) for any of the dependent variables.

**Table 4:** Difference in differences results.

	Likelihood of Purchase		Expected Taste		Brand Liking		Brand Trust	
	Image Removal Only	Image Removal & Name Change	Image Removal Only	Image Removal & Name Change	Image Removal Only	Image Removal & Name Change	Image Removal Only	Image Removal & Name Change
	(1)	(2)	(3)	(4)	(5)	(6)	(7)	(8)
**Post** (*β*)	-0.63**	-2.52***	-0.38**	-1.60***	-0.22	-1.30***	-0.04	-0.94***
	(0.26)	(0.27)	(0.17)	(0.21)	(0.18)	(0.19)	(0.18)	(0.19)
**Racism Info** (*γ*_1_)	0.30	0.00	-0.07	0.14	-0.03	-0.06	-0.20	-0.02
	(0.26)	(0.27)	(0.17)	(0.21)	(0.18)	(0.19)	(0.18)	(0.19)
**Racism & Donation Info** (*γ*_2_)	-0.17	-0.11	-0.14	0.06	-0.12	-0.21	-0.26	-0.11
	(0.26)	(0.27)	(0.17)	(0.21)	(0.18)	(0.19)	(0.18)	(0.19)
**Post x Racism Info** (*δ*_1_)	-0.14	0.77**	-0.04	0.17	-0.13	0.18	0.08	0.10
	(0.36)	(0.38)	(0.24)	(0.29)	(0.26)	(0.26)	(0.25)	(0.27)
**Post x Racism & Donation Info** (*δ*_2_)	-0.03	0.63*	0.05	0.10	-0.04	0.34	0.07	0.25
	(0.36)	(0.38)	(0.24)	(0.29)	(0.26)	(0.26)	(0.25)	(0.27)
**Constant** (*α*_0_)	7.77***	7.91***	8.91***	8.80***	5.54***	5.63***	5.44***	5.37***
	(0.18)	(0.19)	(0.12)	(0.15)	(0.13)	(0.13)	(0.13)	(0.13)

*Note*: Standard errors in parentheses. Significance is denoted by *, **, *** for 10%, 5%, and 1% levels. Both *Racism Information* and *Racism & Donation Information* are compared to the *Alternative Information* treatment.

For likelihood of purchase, we find that removing the image (column 1) brought a loss of -0.63 in likelihood of purchase and that informing consumers that the removal of the image was done to address racism in the packaging had no effect on likelihood of purchase over the alternative reason. Adding a donation also had no effect. When the brand name was also changed (column 2), we find likelihood of purchase dropped by -2.52. When the name is also changed, we find that informing consumers that the rebranding was done to address racism in the packaging increased likelihood of purchase over the alternative reason by 0.77. Although the information increases likelihood of purchase, the change is not large enough to offset the loss from the brand name change. Similarly, adding a donation also provided a marginally significant increase of 0.63 in likelihood of purchase over the alternative information. Additionally, a post-regression t-test confirms that the Racism & Donation Information treatment did not differ significantly from Racism Information treatment alone.

For expected taste, we find that removing the image of Aunt Jemima (column 3) brought small but significant losses (-0.38). Similarly, removing the image and renaming the brand (column 4) brought larger losses to expected taste (-1.60). We find that information did not have a significant impact on expected taste when either the image was removed or when the brand name was also changed.

Additionally, we find that removing the image of Aunt Jemima was not associated with losses in brand liking or brand trust (columns 5 and 7, respectively), however renaming the brand reduced brand liking and brand trust by -1.30 and -0.94, respectively (columns 6 and 8, respectively). Like expected taste, information did not impact brand liking or brand trust for either extent of rebranding.

### Association between political ideology and main outcomes

**Fig 1:**
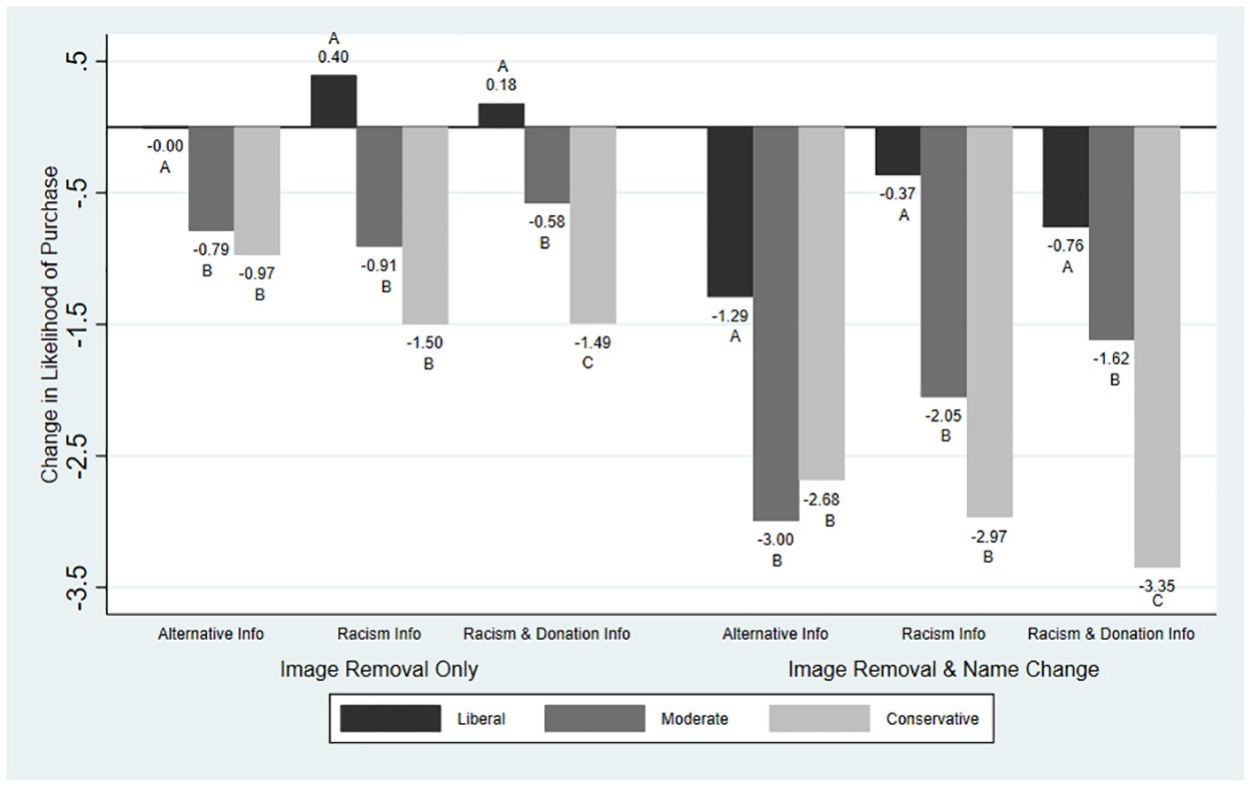
Mean change in likelihood of purchase across treatment groups and political ideology. *Note*: Likelihood of purchase was measured on a scale from 1 (low) to 10 (high). For each treatment, averages with the same letter indicate differences between the political ideologies were not statistically significant.

**Fig 2:**
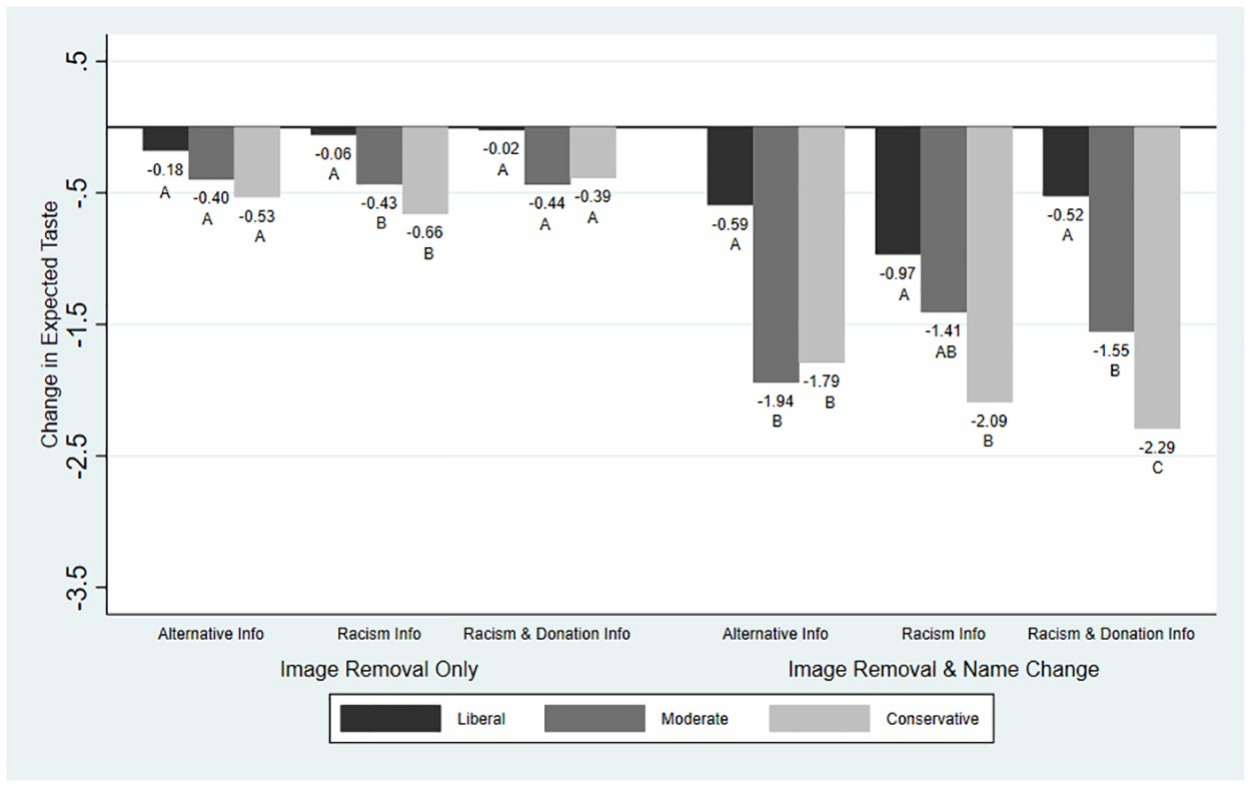
Mean change in expected taste across treatment groups and political ideology. *Note*: Expected taste was measured on a scale from 1 (low) to 10 (high). For each treatment, averages with the same letter indicate differences between the political ideologies were not statistically significant.

**Fig 3:**
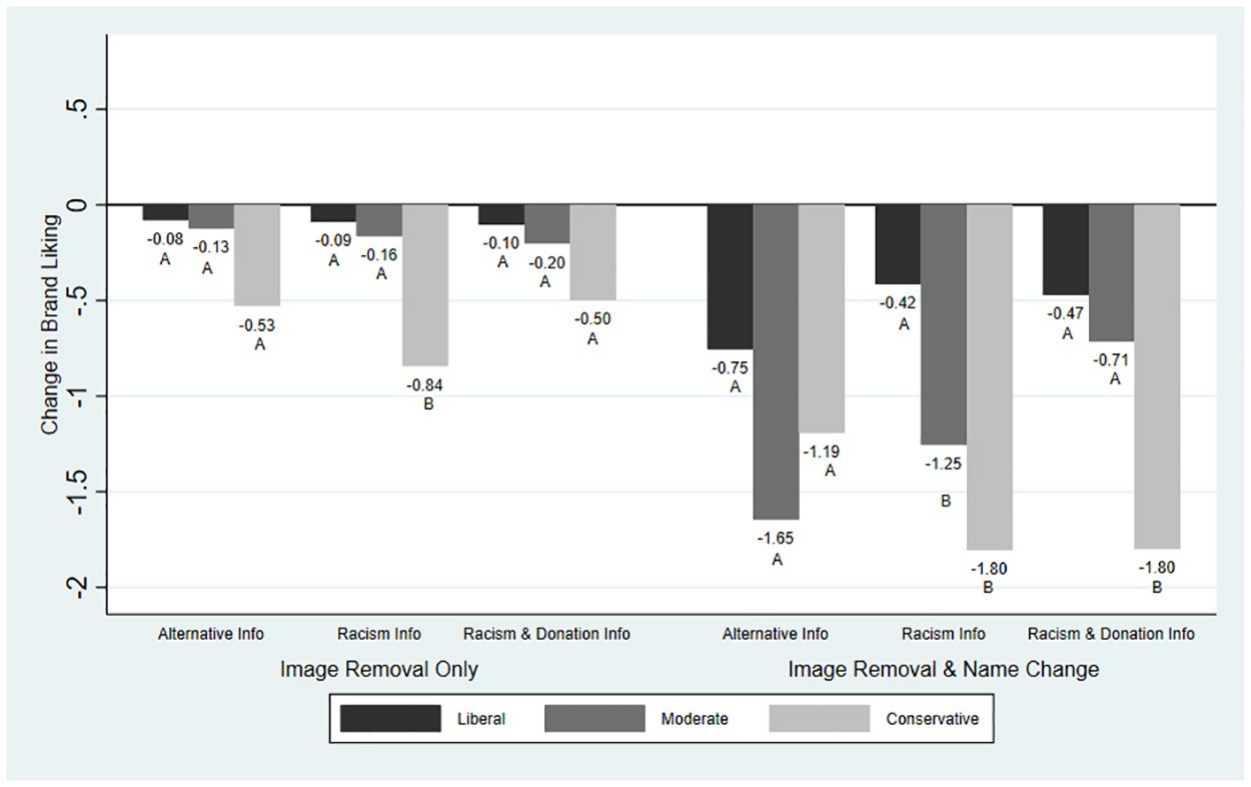
Mean change in brand liking across treatment groups and political ideology. *Note*: Brand liking was measured on a scale from 1 (low) to 7 (high). For each treatment, averages with the same letter indicate differences between the political ideologies were not statistically significant.

**Fig 4:**
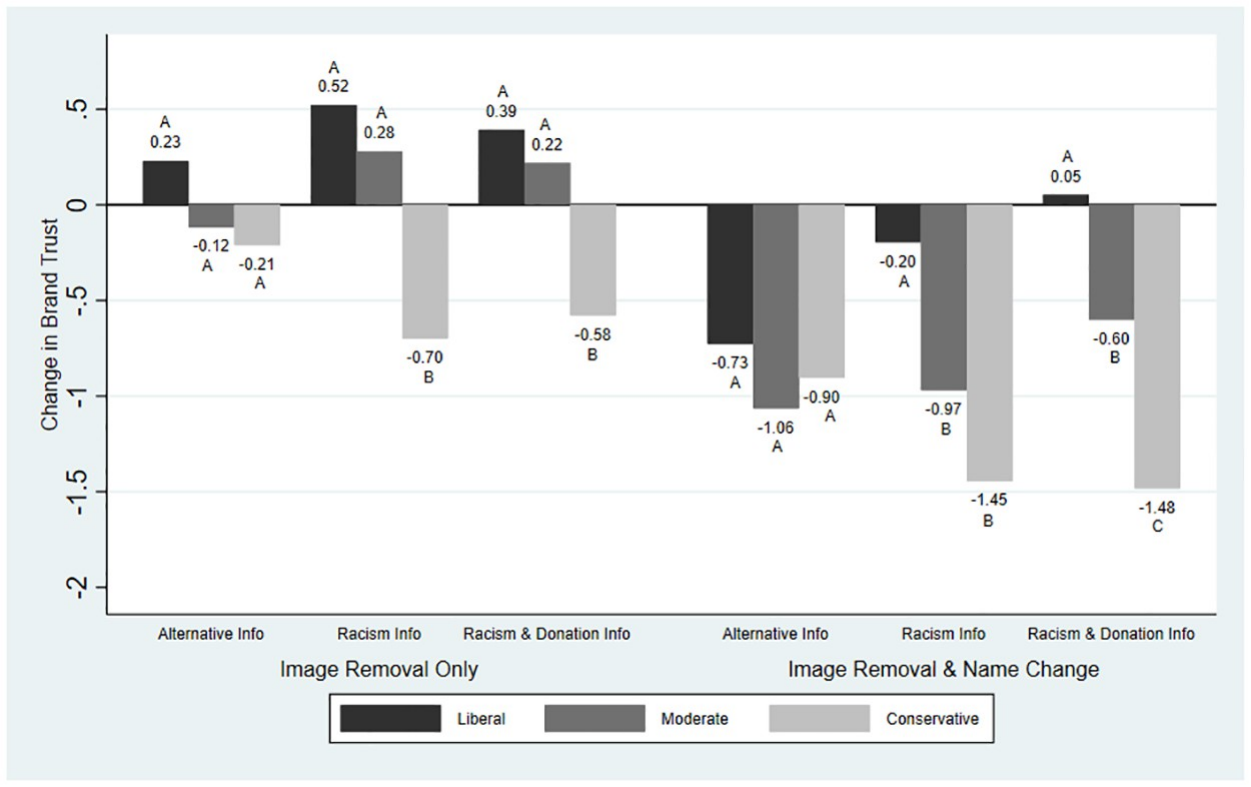
Mean change in brand trust across treatment groups and political ideology. *Note*: Brand trust was measured on a scale from 1 (low) to 7 (high). For each treatment, averages with the same letter indicate differences between the political ideologies were not statistically significant.

Although the impact of information was relatively small or null on average, we find evidence of heterogeneity in the main outcome variables by political ideology. We present these results graphically: Figs 1–4 present the average changes in likelihood of purchase, expected taste, brand liking, and brand trust across political ideologies and treatment groups. The results from the significance testing can be found in S2 Table and are indicated on the graphs.

Fig 1 depicts the changes for likelihood of purchase. Across political ideologies removing the image of Aunt Jemima continued to be associated with smaller changes than renaming the brand. For liberals, removing the image of Aunt Jemima was associated with no change under the Alternative Information and small positive changes under the Racism and Racism & Donation Information. For moderates and conservatives, removing the image of Aunt Jemima was associated with negative effects across information treatments. Changes were more negative for conservatives than moderates. When the company also changed the brand name to Pearl Milling Company, there were larger losses in likelihood of purchase. For liberals, changes in likelihood of purchase were negative, however, Racism Information did seem to provide some buffer. Under the Alternative Information treatment, liberals had a reduction in likelihood of purchase of -1.29, whereas under Racism Information this loss was -0.37 and under Racism & Donation Information this loss was -0.76. For moderates, likelihood of purchase also decreased across information treatments. Here too, we see a potential buffering effect, as the Alternative Information treatment is the most negative (-3.00) compared to Racism Information (-2.05) and Racism & Donation Information (-1.62). Conservatives’ likelihood of purchase decreased across information treatments, between -2.68 and -3.35. Racism Information did not provide a positive buffering effect for conservatives; rather, it appears to have a negative impact. Overall, we find that liberals’ changes in likelihood of purchase differed significantly from both moderates and conservatives across all six treatments and conservatives’ changes in likelihood of purchase differed from moderates under both Racism & Donation information treatments.

Fig 2 depicts changes in expected taste across political ideology and treatment groups. When only the image was removed, expected taste saw no significant differences across political ideology for either Alternative Information or Racism & Donation Information and significant differences across Racism Information. Expected taste decreased more when the brand name was also changed. Racism Information and Racism & Donation Information were associated with increases in expected taste for liberals and moderates and decreases in conservatives compared with the Alternative Information. Changes in expected taste were significantly different across political ideology under all three information treatments.

Fig 3 presents the changes for brand liking. Removing the image of Aunt Jemima brought little change across political ideologies. Differences between political ideologies were not significant for Alternative Information or Racism & Donation Information but differed significantly under Racism Information alone. When the brand name changed to Pearl Milling Company, reductions in brand liking were larger across political ideology and information treatments. Liberals had the smallest losses, ranging between -0.42 (for Racism Information) and -0.75 (for Alternative Information). Moderates had the most negative response to the alternative information (average loss of -1.65), though information on racism provided some buffer, with an average loss of -1.25 and -0.71 for the Racism Information and Racism & Donation Information treatments, respectively. Conservatives had large reductions in brand liking for all treatments, however, the Racism Information further reduced brand liking. The Alternative Information produced an average loss of -1.19 while the average loss was -1.80 under both the Racism Information and Racism & Donation Information treatments. We find that changes in brand liking did not differ significantly across political ideologies under Alternative Information but differed significantly under the Racism Information and Racism & Donation Information treatments.

Fig 4 shows the changes in brand trust across political ideology and treatment group. Removing the image alone was associated with small positive changes for liberals across treatments and for Racism Information and Racism & Donation Information for moderates. The image removal was associated with negative changes for conservatives. Changes in brand trust were significantly different across political ideology under Racism Information and Racism & Donation Information but were not significantly different under Alternative Information. When the brand name was also changed under the Alternative Information, brand trust was reduced by -0.73, -1.06, and -0.90 by liberals, moderates, and conservatives, respectively. Racism Information and Racism & Donation Information provided buffers for liberals. Racism & Donation Information provided a greater buffer for moderates’ change in brand trust compared to Racism Information alone. Similar to brand liking, the Racism Information and Racism & Donation Information decreased brand trust more than the Alternative Information for conservatives. We find that changes in brand trust were significantly different across political ideology when the information treatment was Racism Information or Racism & Donation Information but did not differ significantly across political ideology under Alternative Information.

## Discussion

Brands can serve as indicators of quality and speed up consumer decision making. Consumers can become quite brand loyal and often are willing to pay a premium for their favorites. Rebranding is a large decision for companies, even in the best of times. Recently, some food companies rebranded their products to remove racist images from their packaging and change racist brand names. We investigate consumer responses to these rebranding efforts and assess whether companies can mitigate some of the losses from rebranding by informing consumers about their reason for rebranding.

Using the case of Aunt Jemima, we find that removing the image from the packaging was associated with a relatively small reduction in likelihood of purchase, approximately 8%. We find that removing the image also changes how consumers expect the product will taste, which may indicate that consumers believe the product itself has also been changed—a point discussed by Miller, Stanko, and Diallo [10]. Removing the image did not impact average brand liking or brand trust. Informing consumers that the image removal was done to address racism did not buffer the loss in likelihood of purchase or expected taste over an alternative reason. Adding a donation, similarly, did not buffer the losses.

We find that changing the brand name was associated with much larger shifts. Removing the image and changing the name was associated with a loss in likelihood of purchase of approximately 32%. Expected taste, brand liking, and brand trust also experienced larger reductions than with image removal alone. This is in keeping with the rebranding literature, which has noted that renaming a brand can potentially reduce the benefits that come from brand equity [12]. Additionally, consumers often prefer familiar images, which translates to reduced liking for unfamiliar images (e.g., [8]). Informing consumers that the renaming was done to address racism *did* provide some buffer for likelihood of purchase, mitigating about 10 percent of the loss in likelihood of purchase, but likelihood of purchase was still reduced overall. Informing consumers that the rebranding was done to address racism did not provide a buffer for losses in expected taste, brand liking, or brand trust. Additionally, we find that informing consumers about the company’s donation did not add any additional buffer over noting the change was being done to address racism alone. Additional research is needed to understand consumer perceptions of donations during similar rebranding efforts.

Overall, we find that responses to the rebranding efforts differed significantly across political ideologies, and although informing consumers the rebranding was done to address racism provided small or null impacts on average, we find that responses were quite heterogeneous. Liberal consumers responded most positively to the rebranding and conservative consumers responded most negatively. We also find that Racism Information and Racism & Donation Information seemed to act as a positive buffer over the alternative information for moderates and liberals. However, we find that the information seemed to work in reverse for conservatives, with Racism Information and Racism & Donation Information bringing larger losses than Alternative Information. Consumers across different political ideologies may respond differently to the reason for rebranding for a variety of reasons. For example, the value consumers receive from the product from engaging in political consumerism or from “warm glow” could differ across political ideologies. Additionally, there are large differences across political ideologies both in how consumers feel about calling out potentially offensive content and the Black Lives Matter movement associated with the roots of the rebranding effort. Our results are consistent with previous literature, which has found differences in consumer responses to politically divisive brand actions across political ideologies (e.g., [15]). Here, we find that changes in likelihood of purchase, expected taste, brand liking, and brand trust differed across political ideology and treatment.

### Limitations

While this paper sheds light on consumer responses to rebranding to address racism, some limitations should be acknowledged. First, although likelihood of purchase is a useful initial investigation, it is not a measure of actual behavior, and thus, could suffer from hypothetical bias. Second, every participant in our study was made aware that Aunt Jemima had rebranded moments before their evaluation, which represents a departure from a true customer experience. More likely, customers are either unaware of the rebranding or learned of the rebranding at an earlier time. By structuring our experiment this way, we investigate the impact of the rebranding and the reason for rebranding beyond the impacts of brand recognition, however, the impact of brand recognition could affect the magnitude of the outcomes in the grocery aisle. For example, as the information treatment made it clear that the original and rebranded product were the same brand, the impact of rebranding could be understated as it would not capture the impact of a customer that does not recognize the new packaging, which seems likely in the case of renaming the brand. Third, although our sample is recruited to match the US population in terms of sex, age, income, and census region, online panels are not random samples and are not fully representative of the US population. Together, these limitations underscore the need for additional research to evaluate actual behavior following rebranding to address racism.

Similarly, we use an actual product, Aunt Jemima pancake mix, which allowed us to hold a variety of characteristics constant and most closely reflect an actual rebranding effort. However, by using a real product rather than a hypothetical one, we had to accept that the impact of the information treatments would be imperfect–as some participants in each treatment group will have heard about the rebranding before the survey. The impact of the Racism Information, for example, could then be underestimated, as there will be some participants with previous knowledge randomly assigned to the alternative information treatment, or overestimated if participants inflate their responses to signal approval or disapproval of the change. To check that our results were not driven solely by prior knowledge, we re-ran the analysis using only those who indicated they were not very familiar with the topic beforehand (see S3 Table). We find that the impact of rebranding remained relatively consistent for those who were less familiar with the case. We find that the image removal alone was less impactful for this group, with Image Removal Only having no impact on likelihood of purchase or expected taste. Whereas this group reacted very similarly to when the name was also changed, for example, likelihood of purchase drops by -2.58. Information again has null or small impacts for less familiar consumers. We again find a Racism Information partially mitigates the losses in likelihood of purchase, however, Racism & Donation Information was not associated with a change for this group. In the future, research using fictitious products, or perhaps a relatively unknown product, could confirm these results.

Finally, this is a case and there are likely to be important differences between Aunt Jemima’s pancake mix and their rebranding effort and other products or services undergoing similar changes that impact the outcomes (e.g., a sports team, a smaller brand, a less substantial name change). This limits our ability to extend our results to additional cases. Smaller changes where the brand is still quite recognizable (e.g., removal of an image, less drastic name changes) may require less clarification for consumers.

## Conclusion

Overall, we find that on average, rebranding to address racism is hard for companies to pull off without loss. Removing the image of Aunt Jemima brought losses in likelihood of purchase and expected taste. Renaming the brand brought larger losses in likelihood of purchase and expected taste and reduced brand liking and brand trust. Informing consumers that renaming the brand was done to address racism in the product’s packaging provided some protection, increasing likelihood of purchase some, but did not offset the overall loss from rebranding and did not have an impact on losses in expected taste, brand liking, or brand trust.

This paper evaluates the case of Aunt Jemima pancake mix specifically, and the industry context, timing, and details of the rebranding will impact these results. Additional research is needed to understand how these findings may apply to other contexts and other measures. For example, here were find consumers’ expected taste was reduced from rebranding. Additional research is needed to understand how consumers’ expectations on safety, effectiveness, etc. of products might also be affected. Further, we find that adding a donation had no additional impact over noting the rebranding was done to address racism. Future research could investigate when and why donations would be impactful in these cases. Finally, we find considerable heterogeneity in responses across political ideology. Future research could investigate how different consumer segments respond to this and other divisive brand actions.

## Supporting information

S1 Table Study block stimuli. (DOCX)

S2 Table ANOVA and t-test results. (DOCX)

S3 Table Robustness check. (DOCX)

S1 File Survey data. (CSV)

S2 File Stata code. (DO)

S3 File Code book. (DOCX)
