# Supplementary material for: Consumer responses to rebranding to address racism
Source: PLoS One. 2023 Feb 8;18(2):e0280873. doi: 10.1371/journal.pone.0280873 (PMC9907823; doi:10.1371/journal.pone.0280873)
Supplement: S1 Table — (DOCX) [file pone.0280873.s001.docx]

**Supporting Information:**

**Table S1. Study Block Stimuli**

|  | **Image Removal Only** | **Image Removal & Name Change** |
| --- | --- | --- |
| **Racism Info** | *Below is an excerpt from* *AdWeek, a news source that writes about brands and advertising. Please read it carefully.*  Pepsi Co. announced that they rebranded Aunt Jemima pancake mix to address racism in their brand and packaging. In the new packaging they removed the image of Aunt Jemima. | *Below is an excerpt from AdWeek, a news source that writes about brands and advertising. Please read it carefully.*  Pepsi Co. announced that they rebranded Aunt Jemima pancake mix to address racism in their brand and packaging. In the new packaging they removed the image of Aunt Jemima and renamed the brand Pearl Milling Company. |
| **Racism & Donation Info** | *Below is an excerpt from AdWeek, a news source that writes about brands and advertising. Please read it carefully.*  Pepsi Co. announced that they rebranded Aunt Jemima pancake mix to address racism in their brand and packaging. In the new packaging they removed the image of Aunt Jemima. They also pledged $5 million to support the black community. | *Below is an excerpt from AdWeek, a news source that writes about brands and advertising. Please read it carefully.*  Pepsi Co. announced that they rebranded Aunt Jemima pancake mix to address racism in their brand and packaging. In the new packaging they removed the image of Aunt Jemima and renamed the brand Pearl Milling Company. They also pledged $5 million to support the black community. |
| **Alternative Info** | *Below is an excerpt from AdWeek, a news source that writes about brands and advertising. Please read it carefully.*  Pepsi Co. announced that they rebranded Aunt Jemima pancake mix to increase interest in their brand and packaging. In the new packaging they removed the image of Aunt Jemima. | *Below is an excerpt from AdWeek, a news source that writes about brands and advertising. Please read it carefully.*  Pepsi Co. announced that they rebranded Aunt Jemima pancake mix to increase interest in their brand and packaging. In the new packaging they removed the image of Aunt Jemima and renamed the brand Pearl Milling Company. |

Note: Information treatments included images of the original and rebranded product.
