# Supplementary material for: Consumer responses to rebranding to address racism
Source: PLoS One. 2023 Feb 8;18(2):e0280873. doi: 10.1371/journal.pone.0280873 (PMC9907823; doi:10.1371/journal.pone.0280873)
Supplement: S2 Table — (DOCX) [file pone.0280873.s002.docx]

**Supporting Information:**

**Table S2: Differences in change of likelihood of purchase, expected taste, brand liking, and brand trust across political**

**S2A: Change in Likelihood of Purchase**

| **Image Removal Only** | **Liberals** | **Moderates** | **Conservatives** | **Prob > F** | **H0: Liberal = Conservative** | **H0: Liberal = Moderate** | **H0: Moderate = Conservative** |
| --- | --- | --- | --- | --- | --- | --- | --- |
| **Alternative Info** | 0.00 | -0.79 | -0.97 | 0.05 | 0.02 | 0.05 | 0.63 |
| **Racism Info** | 0.40 | -0.91 | -1.50 | 0.00 | 0.00 | 0.00 | 0.18 |
| **Racism & Donation Info** | 0.18 | -0.58 | -1.49 | 0.00 | 0.00 | 0.05 | 0.04 |
|  |  |  |  |  |  |  |  |
| **Image Removal & Name Change** | **Liberals** | **Moderates** | **Conservatives** | **Prob > F** | **H0: Liberal = Conservative** | **H0: Liberal = Moderate** | **H0: Moderate = Conservative** |
| **Alternative Info** | -1.29 | -3.00 | -2.68 | 0.01 | 0.02 | 0.00 | 0.54 |
| **Racism Info** | -0.37 | -2.05 | -2.97 | 0.00 | 0.00 | 0.00 | 0.12 |
| **Racism & Donation Info** | -0.76 | -1.62 | -3.35 | 0.00 | 0.00 | 0.08 | 0.00 |

Note: Results from ANOVA test (Prob > F) indicate whether there are differences across political ideology for each treatment. For those with significant differences across political ideology, we use t-tests to compare each pair of political ideology (H0: Liberal = Conservative; H0: Liberal = Moderate; H0: Moderate=Conservative).

**S2B: Change in Expected Taste**

| **Image Removal Only** | **Liberals** | **Moderates** | **Conservatives** | **Prob > F** | **H0: Liberal = Conservative** | **H0: Liberal = Moderate** | **H0: Moderate = Conservative** |
| --- | --- | --- | --- | --- | --- | --- | --- |
| **Alternative Info** | -0.18 | -0.40 | -0.53 | 0.42 |  |  |  |
| **Racism Info** | -0.06 | -0.43 | -0.66 | 0.09 | 0.03 | 0.10 | 0.38 |
| **Racism & Donation Info** | -0.02 | -0.44 | -0.39 | 0.28 |  |  |  |
|  |  |  |  |  |  |  |  |
| **Image Removal & Name Change** | **Liberals** | **Moderates** | **Conservatives** | **Prob > F** | **H0: Liberal = Conservative** | **H0: Liberal = Moderate** | **H0: Moderate = Conservative** |
| **Alternative Info** | -0.59 | -1.94 | -1.79 | 0.01 | 0.01 | 0.00 | 0.71 |
| **Racism Info** | -0.97 | -1.41 | -2.09 | 0.08 | 0.02 | 0.27 | 0.16 |
| **Racism & Donation Info** | -0.52 | -1.55 | -2.29 | 0.00 | 0.00 | 0.01 | 0.10 |

Note: Results from ANOVA test (Prob > F) indicate whether there are differences across political ideology for each treatment. For those with significant differences across political ideology, we use t-tests to compare each pair of political ideology (H0: Liberal = Conservative; H0: Liberal = Moderate; H0: Moderate=Conservative).

**S2C: Change in Brand Liking**

| **Image Removal Only** | **Liberals** | **Moderates** | **Conservatives** | **Prob > F** | **H0: Liberal = Conservative** | **H0: Liberal = Moderate** | **H0: Moderate = Conservative** |
| --- | --- | --- | --- | --- | --- | --- | --- |
| **Alternative Info** | -0.08 | -0.13 | -0.53 | 0.30 |  |  |  |
| **Racism Info** | -0.09 | -0.16 | -0.84 | 0.02 | 0.01 | 0.80 | 0.02 |
| **Racism & Donation Info** | -0.10 | -0.20 | -0.50 | 0.37 |  |  |  |
|  |  |  |  |  |  |  |  |
| **Image Removal & Name Change** | **Liberals** | **Moderates** | **Conservatives** | **Prob > F** | **H0: Liberal = Conservative** | **H0: Liberal = Moderate** | **H0: Moderate = Conservative** |
| **Alternative Info** | -0.75 | -1.65 | -1.19 | 0.12 |  |  |  |
| **Racism Info** | -0.42 | -1.25 | -1.80 | 0.01 | 0.01 | 0.02 | 0.21 |
| **Racism & Donation Info** | -0.47 | -0.71 | -1.80 | 0.00 | 0.00 | 0.48 | 0.00 |

Note: Results from ANOVA test (Prob > F) indicate whether there are differences across political ideology for each treatment. For those with significant differences across political ideology, we use t-tests to compare each pair of political ideology (H0: Liberal = Conservative; H0: Liberal = Moderate; H0: Moderate=Conservative).

**S2D: Change in Brand Trust**

| **Image Removal Only** | **Liberals** | **Moderates** | **Conservatives** | **Prob > F** | **H0: Liberal = Conservative** | **H0: Liberal = Moderate** | **H0: Moderate = Conservative** |
| --- | --- | --- | --- | --- | --- | --- | --- |
| **Alternative Info** | 0.23 | -0.12 | -0.21 | 0.43 |  |  |  |
| **Racism Info** | 0.52 | 0.28 | -0.70 | 0.00 | 0.00 | 0.49 | 0.01 |
| **Racism & Donation Info** | 0.39 | 0.22 | -0.58 | 0.02 | 0.02 | 0.61 | 0.02 |
|  |  |  |  |  |  |  |  |
| **Image Removal & Name Change** | **Liberals** | **Moderates** | **Conservatives** | **Prob > F** | **H0: Liberal = Conservative** | **H0: Liberal = Moderate** | **H0: Moderate = Conservative** |
| **Alternative Info** | -0.73 | -1.06 | -0.90 | 0.77 |  |  |  |
| **Racism Info** | -0.20 | -0.97 | -1.45 | 0.05 | 0.02 | 0.07 | 0.33 |
| **Racism & Donation Info** | 0.05 | -0.60 | -1.48 | 0.00 | 0.00 | 0.08 | 0.03 |

Note: Results from ANOVA test (Prob > F) indicate whether there are differences across political ideology for each treatment. For those with significant differences across political ideology, we use t-tests to compare each pair of political ideology (H0: Liberal = Conservative; H0: Liberal = Moderate; H0: Moderate=Conservative).
