# Supplementary material for: Consumer responses to rebranding to address racism
Source: PLoS One. 2023 Feb 8;18(2):e0280873. doi: 10.1371/journal.pone.0280873 (PMC9907823; doi:10.1371/journal.pone.0280873)
Supplement: S3 Table — (DOCX) [file pone.0280873.s003.docx]

**Supporting Information:**

**Table S3: DID results for likelihood of purchase, expected taste, brand liking, and brand trust from rebranding – removing those who were very familiar with the topic before the survey**

|  | **Likelihood of Purchase** | | **Expected Taste** | | **Brand Liking** | | **Brand Trust** | |
| --- | --- | --- | --- | --- | --- | --- | --- | --- |
|  | **Image Removal Only** | **Image Removal & Name Change** | **Image Removal Only** | **Image Removal & Name Change** | **Image Removal Only** | **Image Removal & Name Change** | **Image Removal Only** | **Image Removal & Name Change** |
| **Post (**$\beta$) | -0.51 | -2.58*** | -0.31 | -1.77*** | -0.16 | -1.31*** | 0.12 | -1.03*** |
|  | (0.35) | (0.36) | (0.24) | (0.29) | (0.24) | (0.25) | (0.23) | (0.24) |
| **Racism Info (**$\gamma_{1})$ | 0.28 | 0.25 | -0.05 | 0.03 | 0.07 | -0.09 | 0.00 | -0.01 |
|  | (0.37) | (0.35) | (0.25) | (0.29) | (0.25) | (0.24) | (0.24) | (0.24) |
| **Racism &** | -0.33 | 0.08 | -0.28 | 0.15 | -0.15 | 0.00 | -0.15 | -0.12 |
| **Donation Info (**$\gamma_{2}$) | (0.36) | (0.35) | (0.24) | (0.28) | (0.24) | (0.24) | (0.23) | (0.24) |
| **Post x Racism Info (**$\delta_{1}$) | 0.17 | 1.07** | -0.05 | 0.33 | -0.06 | 0.16 | 0.01 | 0.19 |
|  | (0.52) | (0.50) | (0.36) | (0.41) | (0.35) | (0.35) | (0.34) | (0.34) |
| **Post x Racism &** | 0.07 | 0.77 | -0.07 | 0.25 | 0.08 | 0.36 | (0.02 | 0.52 |
| **Donation Info (**$\delta_{1}$) | (0.51) | (0.49) | (0.35) | (0.40) | (0.34) | (0.34) | (0.33) | (0.34) |
| **Constant (**$\alpha_{0}$) | 7.25*** | 7.31*** | 8.57*** | 8.52*** | 5.30*** | 5.41*** | 5.18*** | 5.19*** |
|  | (0.25) | (0.25) | (0.17) | (0.20) | (0.17) | (0.17) | (0.16) | (0.17) |
