# Supplementary material for: Consumer responses to rebranding to address racism
Source: PLoS One. 2023 Feb 8;18(2):e0280873. doi: 10.1371/journal.pone.0280873 (PMC9907823; doi:10.1371/journal.pone.0280873)
Supplement: S3 File — (DOCX) [file pone.0280873.s006.docx]

| **Dependent Variables** |  |
| --- | --- |
| BL | Brand Liking (reshaped) |
| BL_1 | Brand Liking (prior to rebranding: t0) |
| BL_2 | Brand Liking (post rebranding: t1) |
| dBL | Change in Brand Liking |
| BT | Brand Trust (reshaped) |
| BT_1 | Brand Trust (prior to rebranding: t0) |
| BT_2 | Brand Trust (post rebranding: t1) |
| dBT | Change in Brand Trust |
| ET | Expected Taste (reshaped) |
| ET_1 | Expected Taste (prior to rebranding: t0) |
| ET_2 | Expected Taste (post rebranding: t1) |
| dET | Change in Expected Taste |
| LOP | Likelihood of Purchase (reshaped) |
| LOP_1 | Likelihood of Purchase (prior to rebranding: t0) |
| LOP_2 | Likelihood of Purchase (post rebranding: t1) |
| dLOP | Change in Likelihood of Purchase |
| **Treatment Variables** |  |
| group | Treatment Group number  1 = Racism; Moderate  2 = Racism; Radical  3 = Racism & Donation; Moderate  4 = Racism & Donation; Radical  5 = Interest; Moderate  6 = Interest; Radical |
| GRP1 | Group dummy |
| GRP2 | Group dummy |
| GRP3 | Group dummy |
| GRP4 | Group dummy |
| GRP5 | Group dummy |
| GRP6 | Group dummy |
| extent | Dummy for extent of rebranding  0 = Image Removal Only Rebranding  1 = Image Removal and Name Change |
| info | Information treatment assigned  1= racism info  2 = donation info  0 = alt info (interest) |
| info_racism | Dummy for racism info treatment  1= racism info  0 = o.w. |
| info_donation | Dummy for racism & donation info treatment  1 = racism & donation info  0 = o.w. |
| period | Dummy for reshaping data  1 = original packaging (t0)  2 = rebranded packaging (t1) |
| post | Dummy for period  0 = original packaging (t0)  1 = rebranded packaging (t1) |
| racism_post | Interaction between info_racism and post |
| donation_post | Interaction between info_donation and post |
| **Participant Characteristics** |  |
| sex | Sex |
| age | Age  1 = 18-24  2 = 25-34  3 = 35-44  4 = 45-54  5 = 55-64  6 = 65-74  7 = 75+ |
| region | Region  1 = North East  2 = Midwest  3 = South  4 = West |
| race | Race  1 = American Indian or Alaska Native  2 = Asian  3 = Black or African American  4 = Native Hawaiian or other Pacific Islander  5 = White  6 = Other |
| hisp | Ethnicity  0 = Not Hispanic/Latino  1 = Hispanic/Latino |
| edu | Education  1 = less than high school  2 = high school/ged  3 = some college  4 = associates or technical degree  5 = bachelors  6 = graduate or professional degree |
| college | Simplification of education variable  0 = less than bachelors  1= bachelors or higher |
| inc | Annual Household Income  1 = Less than $25,000  2 = $25,000 to $49,999  3 = $50,000 to $74,999  4 = $75,000 to $99,999  5 = $100,000 to $124,999  6 = $125,000 to $149,999  7 = $150,000 or more |
| pol | Political ideology  1 = Very liberal  2 = Liberal  3 = Moderate  4 = Conservative  5 = Very conservative |
| pol2 | Simplification of political ideology variable  liberal = 1  moderate = 2  conservative = 3 |
| know | Before participating in this survey, how familiar were you with the rebranding of Aunt Jemima's pancake mix?  1 = not familiar at all  2 = slightly familiar  3 = moderately familiar  4 = very familiar  5 = extremely familiar |
